# Supplementation with Standardized Green/Black or White Tea Extracts Attenuates Hypertension and Ischemia-Reperfusion-Induced Myocardial Damage in Mice Infused with Angiotensin II

**DOI:** 10.3390/antiox14010047

**Published:** 2025-01-03

**Authors:** Mario de la Fuente-Muñoz, Marta Román-Carmena, Sara Amor, María C. Iglesias-de la Cruz, Patricia Martorell, Sonia Guilera-Bermell, Reme García Bou, Antonio M. Inarejos-García, Ángel L. García-Villalón, Miriam Granado

**Affiliations:** 1 Departamento de Fisiología, Facultad de Medicina, Universidad Autónoma de Madrid, 28029 Madrid, Spain; mario.delafuente@inv.uam.es (M.d.l.F.-M.); sara.amor@uam.es (S.A.); mc.cruz@uam.es (M.C.I.-d.l.C.); angeluis.villalon@uam.es (Á.L.G.-V.); 2 Nutrition Archer Daniels Midland (ADM) Health & Wellness, Biopolis S. L. Parc Cientific, Universitat de València, 46980 Paterna, Spain; patricia.martorell@adm.com; 3 R&D Department of Functional Extracts, ADM, 46740 Valencia, Spain; sonia.guilera@adm.com (S.G.-B.); reme.garcia@adm.com (R.G.B.); antonio.inarejos@adm.com (A.M.I.-G.); 4 CIBER Fisiopatología de la Obesidad y Nutrición, Instituto de Salud Carlos III, 28029 Madrid, Spain

**Keywords:** cardiovascular, hypertension, angiotensin II, green tea, black tea, white tea, antioxidant, inflammation

## Abstract

Arterial hypertension has a high prevalence in the population and is considered both a cardiovascular disease and an important risk factor for the development of other cardiovascular diseases. Tea consumption shows antihypertensive effects due to its composition in terms of bioactive substances such as flavan-3-ols and xanthines. The aim of this study was to assess the possible beneficial effects of two tea extracts, one of white tea (ADM^®^ White Tea; WTE) and another one composed of a mixture of black tea and green tea (ADM^®^ Tea Complex; CTE), on the cardiovascular alterations induced by angiotensin II (AngII) infusion in mice. For this purpose, four groups of C57BL/6J male mice were used: (1) mice fed on a standard diet for 8 weeks and infused with saline for the last 4 weeks (controls); (2) mice fed on a standard diet for 8 weeks and infused with AngII for the last 4 weeks (AngII); (3) mice fed on a standard diet supplemented with 1.6% WTE and infused with AngII for the last 4 weeks (AngII + WTE); (4) mice fed on a standard diet supplemented with 1.6% TC and infused with AngII for the last 4 weeks (AngII + CTE). Both tea extracts exerted anti-inflammatory and antioxidant effects in arterial tissue and reduced AngII-induced endothelial dysfunction in aorta segments. Moreover, supplementation with WTE or CTE reduced the Ang-II-induced overexpression of AT1R and increased AngII-induced downregulation of AT2R in arterial tissue. However, only supplementation with CTE significantly increased the circulating levels of angiotensin 1-7 and reduced systolic blood pressure. In the heart, supplementation with both tea extracts attenuated AngII-induced cardiac hypertrophy and reduced ischemia-reperfusion-induced oxidative stress and apoptosis in myocardial tissue. In conclusion, supplementation with WTE or CTE attenuates AngII-induced cardiovascular damage through their anti-inflammatory, antioxidant, and antiapoptotic effects. In addition, supplementation with CTE also exerts antihypertensive effects, and so it may constitute an avenue through which to support cardiovascular health.

## 1. Introduction

Currently, more than 30% of the adult population suffers from hypertension, which is considered one of the main causes of premature death in the world [1]. Furthermore, hypertension is one of the main risk factors for the development of other cardiovascular diseases, including coronary heart disease, heart failure, stroke, myocardial infarction, or chronic kidney disease, among others [2]. The most common type of hypertension is essential hypertension (EH), which affects 90% of the patients. EH has a multifactorial origin that involves both genetic and physiological factors such as aging and environmental factors such as a sedentary lifestyle and the consumption of a diet high in salt and low in potassium and calcium [3].

Blood pressure regulation is carried out by a wide variety of physiological systems, among which the renin–angiotensin–aldosterone system (RAAS) stands out. RAAS is responsible for controlling blood volume and vascular tone through a cascade of different peptides and enzymes: renin first synthesizes angiotensin I from angiotensinogen, which is then used by the angiotensin-converting enzyme (ACE) to synthesize angiotensin II (AngII) [4]. The effects of AngII are produced through its binding to AngII type 1 (AT1R) and AngII type 2 (AT2R) receptors. At the vascular level, the main action of AngII upon binding to AT1R is the vasoconstrictor effect. In pathological conditions, the hyperactivation of RAAS produces deleterious effects in the cardiovascular system, which ultimately leads to the development of hypertension and other cardiovascular alterations [5].

Oxidative stress plays a fundamental role in AngII-induced tissue damage. In the blood vessels, the main source of ROS is NADPH oxidases, especially the isoforms NOX 1, NOX 2, and NOX 4, which are found in the cytosol of endothelial and smooth muscle cells [6]. In pathological conditions, AngII causes the sustained activation of NADPH oxidases, leading to the increased production of reactive oxygen species (ROS) [7] such as superoxide anion and hydrogen peroxide which, in the long term, may not be completely neutralized by the cellular antioxidant defenses, resulting in an imbalance of the redox homeostasis. In this context, the excess of ROS leads to structural and functional alterations such as vascular remodeling [8], endothelial dysfunction, and altered vasorelaxation due to the decreased activation of the endothelial nitric oxide synthase enzyme (eNOS) and decreased nitric oxide (NO) availability [9,10].

Besides being the main regulator of vascular tone, NO is also a mediator of the inflammatory response. Particularly, it exerts antiproliferative functions and inhibits leukocyte adhesion in the arterial wall. In the context of increased oxidative stress, the reduced bioavailability of NO produces an increase in the expression of adhesion molecules in the vascular endothelium, which allows the migration and accumulation of inflammatory cells such as macrophages to the vascular wall. These accumulated cells in the subendothelial space of the arteries release proinflammatory cytokines that trigger a proinflammatory state that further contributes to the development of vascular remodeling and endothelial dysfunction [9,11]. The latter is characterized by an alteration in the equilibrium between endothelium-derived relaxing and constricting factors, favoring the development of cardiovascular diseases such as hypertension [12].

In addition, the maintained overstimulation of RAAS not only produces alterations in the blood vessels but also in the heart. As in vascular tissue, the main sources of reactive oxygen species in the myocardium are NOX-2 and NOX-4 enzymes [13]. In the heart, the AngII-induced increase in ROS leads to alterations in cardiomyocyte differentiation and proliferation, and stimulates cardiomyocyte apoptosis which, in the long term, contributes to the development of cardiac dysfunction, hypertrophy, and failure [14].

**Scheme 1:**
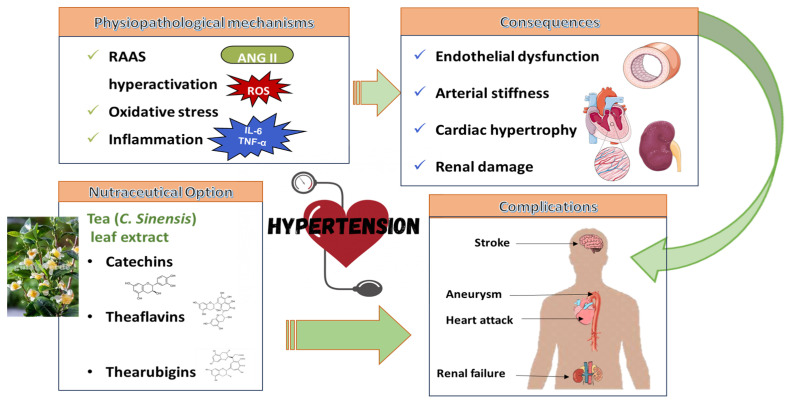
Mechanisms and complications of hypertension and tea supplementation as a nutraceutical option to support cardiovascular health and particularly the cardiovascular alterations associated with hypertension.

Since oxidative stress plays a key role in the development of hypertension and its cardiovascular-related alterations (Scheme 1), substances with antioxidant effects have received attention as possible candidates for its management [15]. In this regard, different studies have shown that diets rich in fruits and vegetables are able to reduce blood pressure thanks to their content in antioxidant compounds such as vitamin C and E, carotenoids, and polyphenols, among others [16,17,18]. This has prompted the use of foods and supplements rich in antioxidant substances as complementary options to conventional pharmacological antihypertensive treatments [19]. Among these foods/supplements is tea, a commonly consumed beverage that is reported to exert beneficial effects on cardiovascular function by reducing blood lipid levels, attenuating inflammation, inhibiting oxidative stress, enhancing endothelial function, alleviating ischemia/reperfusion injury, and protecting cardiomyocyte function [20]. These effects are attributable to the presence of several bioactive compounds including catechins, theaflavins, thearubigins, and phenolic compounds [21]. The polyphenols present in tea have a strong capacity to reduce the production of free radicals, either by eliminating them directly or indirectly, by regulating the activity of NADPH oxidases, or by regulating the expression of antioxidant enzymes such as superoxide dismutase or glutathione peroxidase [22,23].

Numerous experimental studies in animals have shown that tea polyphenols significantly reduce the production of ROS, resulting in increased activation of eNOS in endothelial cells and NO bioavailability, which promotes vasodilatation [24,25]. Moreover, there are also some studies in humans in which regular tea consumption lowers systolic and diastolic blood pressure, suggesting the potential role of tea in the management of hypertension [26,27,28]. However, the beneficial cardiovascular effects found in humans are, in general, modest and less satisfactory than those described in animal models [29].

In a previous study, our group demonstrated that supplementation with two standardized tea extracts—one made of white tea, known as ADM^®^ White Tea (WTE), and another made of green and black tea, known as ADM^®^ Complex Tea Extract (CTE)—is able to improve endothelial function and reduce blood pressure in mice with metabolic syndrome due to their antioxidant and anti-inflammatory effects [30]. However, since supplementation with tea extracts, especially with CTE, also produced a significant improvement in insulin resistance and lipid profile [31], it is not completely clear if the beneficial effects on blood pressure were due to a direct antihypertensive effect or were just the result of an improvement in the general metabolic state of the animals. For this reason, the aim of this study was to check whether these same standardized tea extracts, WTE and CTE, also exert beneficial effects on blood pressure in a conventional experimental model of hypertension induced by AngII infusion in mice. Our hypothesis is that supplementation with both tea extracts may exert beneficial effects on myocardial and arterial tissues, attenuating the cardiovascular damage induced by AngII infusion.

## 2. Materials and Methods

### 2.1. Commercial Tea Extracts

For this study, two different commercial tea powdered extracts, commercialized by the company Archer Daniels Midland Co. (ADM) Wild Valencia (Carcaixent, Spain), were employed. The first consists of a proprietary blend of green and black tea leaves, standardized to total flavan-3-ols (monomeric and theaflavins) and methylxanthines (caffeine, theobromine, and theophylline). This is named ADM^®^ Complex Tea Extract (CTE). The second one was a white tea extract standardized to monomeric flavan-3-ols and xanthines. This is named ADM^®^ White Tea extract (WTE). The chemical characterization of the two tea extracts was performed via high-performance liquid chromatography (HPLC). The content of the main groups of components (flavan-3-ols, xanthines, theaflavins and gallic acid) present in the extract was reported in a previous study [30].

### 2.2. Antioxidant Characterization of Plasma Samples

The analysis of polar metabolites in plasma samples was performed by HPLC according to the method proposed by Wang et al. [32]. An HPLC device, Shimadzu Nexera XR HPLC 70 MPa, was coupled to a photodiode array detector, SPD-M40 model, and used for chromatographic analysis (Izasa Scientific, Madrid, Spain). Briefly, the extraction process consisted of mixing 120 mL of plasma with 10 mL of ascorbic acid (1 nM). β-Glucuronidase deconjugation was performed via incubation at 37 °C for 1 h. At the end of the reaction, 1 mL of acetonitrile was added. After homogenization and centrifugation, the supernatant was evaporated and redissolved in 50% methanol before HPLC analysis. The HPLC conditions were as follows: Phase A was a 0.1% formic acid solution and Phase B involved acetonitrile. The separation of the flavonoids was performed with a linear gradient. We used 5–15% B from 0 to 45 min, 15–80% B from 45 to 50 min, 80% B from 50 to 53 min, and 5% B from 53 to 70 min. The flow was set to 0.7 mL/min, the oven temperature was set to 40 °C, and the absorbance was monitored at 280 nm through an UV detector.

To assess the antioxidant capacity of plasma samples, the 2,2-diphenyl-1-picrylhydrazyl (DPPH) assay was used. First, plasma samples were deconjugated according to the method described by Wang et al. [32] and then the antioxidant capacity of deconjugated samples was assessed according to the method described by Brand-Williams et al. [33]. Briefly, we used a Trolox ((±)-6-Hydroxy-2,5,7,8-tetramethylchromane-2-carboxylic acid) calibration curve (0.02–0.5 mM) based on methanol and a working solution (0.12 mM) prepared from a stock of DPPH. Then, deconjugated samples in methanol were filtered through a nylon syringe filter (0.45 µm) before performing the spectrophotometric analysis with the nano-spectrophotometer SpectrostarNano (Labtech BMG, Ortenberg, Germany) at 517 nm. Results were expressed as µM Trolox equivalents/µL plasma. A DPPH-synthetic free radical (2,2-diphenil-1-1picrilhidracyl) and (±)-6-Hydroxy-2,5,7,8-tetramethylchromane-2-carboxylic acid (Trolox 97% purity) were purchased from Merck (Tres Cantos, Madrid, Spain).

### 2.3. Analysis of Malondialdehyde (MDA) in Plasma Samples by HPLC

The derivatization of plasma samples was performed according to the method described by Cristina Más-Bargues et al. [34]. Then, the MDA analysis was performed according to the work of Wong et al. [35]. An HPLC device, Shimadzu Nexera XR HPLC 70 MPa, coupled to a photodiode array detector, SPD-M40 (Izasa Scientific, Spain), was used for chromatographic analysis. MDA-bis was purchased from Merck (Tres Cantos, Spain)

### 2.4. In Vivo Study

#### 2.4.1. Animals

For the in vivo study, 31 16-week-old C57/BL6J male mice were housed, 3 per cage, and maintained in climate-controlled quarters under controlled conditions of temperature (22–24 °C) and humidity (50–60%) with a 12 h light cycle. All experiments were conducted according to the European Union Legislation and with the approval of the Animal Care and Ethical Committee of the Community of Madrid (Madrid, Spain) (PROEX 282.2-22).

#### 2.4.2. Treatments

The mice were fed ad libitum for 8 weeks with a standard diet or a diet supplemented with 1.6% of WTE or 1.6% CTE (ResearchDiets Inc., New Brunswick, NJ, USA). After four weeks of treatment, alzet osmotic minipumps (Alza Corp., Cupertino, CA, USA) were subcutaneously implanted under isofluorane anesthesia to infuse either AngII (1.44 mg/Kg/day) or saline for 28 days with an infusion rate of 0.25 µL/h. At this moment, the mice were divided into four experimental groups; mice fed with a standard diet and infused with saline (Chow; n = 7); mice fed with a standard diet and infused with AngII (AngII; n = 8), mice fed with a standard diet supplemented with 1.6% CTE and infused with AngII (AngII + CTE; n = 8); and mice fed with a standard diet supplemented with 1.6% WTE and infused with AngII (AngII + WTE; n = 8).

During treatment, body weight and solid intake were monitored once a week. After 8 weeks of treatment, all animals were sacrificed via an overdose of sodium pentobarbital (100 mg/kg) followed by decapitation. After euthanasia, the blood was collected in tubes with EDTA (1.5 mg/mL) and centrifuged at 3000 rpm for 20 min to obtain plasma. Then, the heart and the aorta were carefully dissected and used for later functional and molecular studies.

#### 2.4.3. Measurement of Systolic Arterial Pressure in Conscious Mice by Plethysmography

Systolic blood pressure was measured twice every week via transmission photoplethysmography using a BP-2000 Blood Pressure Analysis System (Visitech Systems, Apex, NC, USA). For this purpose, the mice were placed on a prewarmed plate at 37 °C and an occlusion cuff was placed at the base of the tail. Each recorded session consisted of 30 accurate inflation–deflation cycles. All mice were habituated to the device at least three days before the measurements.

#### 2.4.4. Plasma Concentrations of Angiotensin 1-7

The plasma concentrations of angiotensin 1-7 (Ang 1-7) were measured by an ELISA kit (ABclonal, Woburn, MA, USA) following the manufacturer’s instructions. Sensitivity and intra-assay variations were 2.2 pg/mL and 6.4%, respectively.

#### 2.4.5. Experiments of Vascular Reactivity

Then, 2 mm thoracic aorta segments was mounted in a 4 mL organ bath for the recording of isometric tension using a PowerLab C data acquisition system (ADInstruments, Colorado Springs, CO, USA). Segments were first equilibrated for 60–90 min and an optimal passive tension of 1 g was applied. After equilibration, potassium chloride (KCl 100 mM, Merck Millipore, Burlington, MA, USA) was added to the organ bath. Those segments unable to contract by at least 0.3 g in response to KCl were discarded.

For the vasodilation studies, the aortic segments were precontracted with the thromboxane analogue U46619 10^−7.5^ M (Sigma-Aldrich, St. Louis, MO, USA). Then, we assessed the vascular response to a single dose of insulin (10^−6^ M) and to cumulative doses of sodium nitroprusside (NTP; 10^−9^–10^−5^ M) and acetylcholine (Ach; 10^−9^–10^−4^ M) in order to test endothelium-independent and endothelium-dependent vasodilation, respectively. The effect of oxidative stress on endothelial function was determined by preincubating some of the thoracic segments with the NADPH inhibitor apocynin (10^−6^ M) for 30 min before examining the Ach dose–response curves. The % of relaxation in response to each ACh and insulin concentrations was calculated with reference to the maximum relaxation (Emax) in response to NTP 10^−5^ M.

#### 2.4.6. Experiments of Heart Perfusion: Langendorff

The heart was immediately dissected, cannulated by the aorta, and mounted in a heart perfusion system (Langendorff). Through this system, the heart was retrogradely perfused with a Krebs–Henseleit physiological solution (115 mM NaCl, 4.6 mM KCl, 1.2 mM KH_2_PO_4_, 1.2 mM MgSO_4_, 2.5 mM CaCl_2_, 25 mM NaHCO_3_ and 11 mM glucose) at a pH of 7.4 and at a constant flow between 12–17 mL/min by a peristaltic pump (Gilson, Middleton, WI, USA), providing a basal perfusion pressure of around 70 mmHg. The coronary perfusion pressure was measured through a lateral pressure transducer connected to the perfusion cannula (Statham Instruments, Los Angeles, CA, USA) and the ventricular pressure was measured by inserting a latex balloon connected to another pressure transducer into the left ventricle. The left ventricular pressure was used to calculate the heart rate and the first derivative of the left ventricular pressure curve (dP/dt) as an index of heart contractility. After a 30 min equilibration period with constant flow, global ischemia was induced by stopping the flow perfusion for 30 min. Afterwards, the hearts were reperfused for 45 min. After ischemia-reperfusion (IR), the hearts were collected and stored at −80 °C for further analysis. The data of cardiac function were recorded using the PowerLab/8e data acquisition system (ADInstruments, Colorado Springs, CO, USA).

#### 2.4.7. Quantitative RT-PCR Assay

The total RNA was extracted from aorta and myocardial tissue using the Tri-Reagent protocol [36]. Then, 1 μg of RNA was reverse-transcribed into cDNA using a high-capacity cDNA RT kit (Applied Biosystems, Foster City, CA, USA). Quantitative real-time PCR was performed by using assay on-demand kits (Applied Biosystems) for each gene. TaqMan Universal PCR Master Mix (Applied Biosystems, Foster City, CA, USA) was used for amplification according to the manufacturer’s protocol via Step One System equipment (Applied Biosystems, Foster City, CA, USA). The mRNA concentrations of interleukin-6 (*Il-6*) (Mm00446190_m1), interleukin-1 beta (*Il-1β*) (Mm00434228_m1), interleukin-10 (*Il-10*) (Mm01288386_m1), tumor necrosis factor-alpha (*TNF-α*) (Mm00443258_m1), monocyte chemoattractant protein (*Mcp-1*) (Mm00441242_m1), glutathione reductase (*Gsr*) (Mm00439154_m1), NADPH oxidase-4 (*Nox-4*) (Mm00479246_m1), NADPH oxidase-1 (*Nox-1*) (Mm00549170_m1), superoxide dismutase 1 (*Sod-1*) (Mm01344233_g1), glutathione peroxidase 3 (*Gpx3*) (Mm00492427_m1), angiotensin receptor type-1 (*At1r*) (Mm00616371_m1), angiotensin receptor type-2 (*At2r*) (Mm01341373_m1), and angiotensin I-converting enzyme 2 (*Ace2*) (Mm01159006_m1) were assessed. Values were normalized to the housekeeping Hypoxanthine Phosphoribosyltransferase 1 (*Hprt1*) (Mm03024075_m1). To determine the relative expression levels, the 2^−ΔΔCT^ method was used [37]. Data are expressed as a percentage of the control group.

#### 2.4.8. Protein Quantification by Western Blot

To quantify the expression of proteins involved in the apoptotic pathways, 100 mg of myocardial tissue was homogenized in 500 μL of RIPA buffer and centrifugated at 14,000 rpm for 20 min at 4 °C. The supernatant was collected, and the total protein content was measured by the Bradford method (Sigma-Aldrich, St. Louis, MO, USA). Then, 10 µg of total protein was separated by electrophoresis on 10% acrylamide SDS gels (Bio-Rad, Hércules, CA, USA) and transferred to polyvinylidene difluoride (PVDF) membranes (Bio-Rad, Hércules, CA, USA). Transfer efficiency was determined by Ponceau red dyeing (Sigma-Aldrich, St. Louis, MO, USA). Membranes were then blocked with tris-buffered saline (TBS) containing 5% (*w*/*v*) non-fat dried milk and incubated with the primary antibodies for Caspase 8 (1:2000; #PA5-20118; Thermo Scientific, Waltham, MA, USA) or Bcl-2 (1:1000; #PA1-30411; Thermo Scientific, Waltham, MA, USA) at 4 °C overnight. Next, membranes were washed with TBS-T and incubated with the secondary antibody conjugated with peroxidase (1:2000; Pierce, Rockford, IL, USA). Peroxidase activity was visualized by chemiluminescence and quantified by densitometry using BioRad Molecular Imager ChemiDoc XRS System (Hércules, CA, USA). For each sample, relative protein expression levels were calculated as a percentage of the control group (Chow).

#### 2.4.9. Histological Analysis

Sections of aorta and heart were fixed in 4% paraformaldehyde. Then, they were dehydrated, embedded in paraffin, and sectioned in slices of 5 μm thickness. To assess the thickness of the tunica media, arterial sections were stained with hematoxylin/eosin.

The vascular production of superoxide anions was assessed using the oxidative fluorescent dye dihydroethidium (DHE, Invitrogen Life Technologies, Carlsbad, CA, USA). Briefly, DHE was dissolved in DMSO (2.5 mM), added to each tissue section, and incubated in a dark and humidified chamber for 30 min at 37 °C. Afterwards, the tissue sections were mounted with Vectashield (Newark, CA, USA). Images were obtained using a confocal microscope (Leica TCS SP2 equipped with a krypton/argon laser, ×40 objective; Leica Microsystems, Wetzlar, Germany). Fluorescence was detected with a 563 nm long-pass filter. For quantification, the mean fluorescence density in the target region was analyzed; two to three rings or regions per animal from each experimental group were sampled and averaged. Data were expressed as a percentage of the signal in arteries from control mice (Chow).

TUNEL staining was used to study apoptosis in cardiac tissue according to the manufacturer’s protocol (Click-iTTM Plus TUNEL Assay; Invitrogen). Samples were observed with a fluorescent laser scanning confocal microscope (Leica TCS SP5 equipped with 40× oil objective; Leica Microsystems S.L.U.) Fluorescence was detected with a 519 nm long-pass filter. Quantitative analysis was performed with ImageJ 1.54 and the results were expressed as the percentage of cells with fragmented DNA vs. the total number of cells. For quantification, three images of different regions per animal were sampled. Data were expressed as a percentage of the signal in arteries from control mice.

### 2.5. Statistical Analysis

The statistical analysis of data was conducted using the GraphPad Prism V.70.4 statistical software (GraphPad Software, Inc., San Diego, CA, USA). All data were expressed as mean values ± standard error of the mean (SEM). Results were analyzed using one-way ANOVA followed by Bonferroni’s post hoc test. A *p* < 0.05 was considered statistically significant.

## 3. Results

### 3.1. Body Weight, Food Intake and Organ Weights

Hypertensive mice treated with WTE or CTE showed a significant reduction in body weight gain after 8 weeks of treatment compared to controls (Table 1; *p* < 0.001 and *p* < 0.01, respectively) and untreated hypertensive mice (Table 1; *p* < 0.001 and *p* < 0.01, respectively) that was associated with a significant decrease in solid food intake (Table 1; *p* < 0.01 for both).

Regarding organ weights, untreated hypertensive mice showed increased weight in the heart (*p* < 0.01) and adrenal glands (*p* < 0.05), as well as decreased liver weight (*p* < 0.05), compared to normotensive mice. Supplementation with both extracts reduced the weights of heart (*p* < 0.05 for both), spleen (*p* < 0.01 for both), epididymal visceral adipose tissue (*p* < 0.01 for WTE and *p* < 0.05 for CTE), retroperitoneal visceral adipose tissue (*p* < 0.01 for both), and kidney (*p* < 0.05 for both) compared to hypertensive mice. In addition, WTE treatment also significantly reduced liver weight (*p* < 0.05) and CTE treatment reduced the weight of lumbar subcutaneous adipose tissue compared to both control and untreated AngII-infused mice (*p* < 0.05).

### 3.2. Cardiac Function

The parameters related to cardiac function, shown in Table 2, were measured before and after subjecting the hearts of mice from the different experimental groups to ischemia reperfusion (IR). Before IR, AngII-infused mice showed increased coronary pressure compared to the normotensive mice group (*p* < 0.05) that was attenuated by WTE supplementation.

No changes in coronary pressure were found among experimental groups after IR. Regarding heart contractility, no significant differences were found among experimental groups before IR. In contrast, hearts from mice supplemented with CTE or WTE showed increased contractility compared to hypertensive mice after IR (*p* < 0.05).

### 3.3. Effects of WTE and CTE on Inflammation and Oxidative Stress in Hearts Subjected to IR

**Figure 1:**
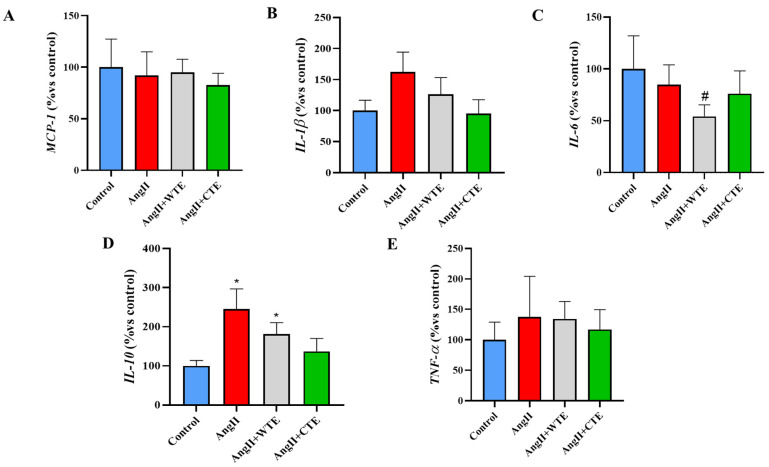
The gene expression of Monocyte Chemotactic Protein-1 (**A**), interleukin-1β (**B**), interleukin-6 (**C**), interleukin-10 (**D**), and tumor necrosis factor α (**E**) in heart of mice fed on a standard chow for 8 weeks and infused with saline for the last 4 weeks (control); mice fed on a standard chow for 8 weeks and infused with AngII for the last 4 weeks (AngII); mice fed on a standard chow supplemented with White Tea Extract for 8 weeks and infused with AngII for the last 4 weeks (AngII + WTE); and mice fed on a standard chow supplemented with Complex Tea Extract for 8 weeks and infused with AngII for the last 4 weeks (AngII + CTE). Data are represented as mean value ± SEM, n = 8–10 mice/group, and expressed as a percentage vs. control; * *p* < 0.05 vs. control; # *p* < 0.05 vs. AngII.

The gene expression of inflammatory markers in myocardial tissue is shown in Figure 1. No significant differences were found in the mRNA levels of inflammatory markers, except for a significant upregulation in the gene expression of *Il-10* in untreated AngII-infused mice and in AngII-infused mice supplemented with WTE (*p* < 0.05 for both). Moreover, WTE supplementation significantly reduced the mRNA levels of the proinflammatory cytokine *Il-6* compared to untreated hypertensive mice (*p* < 0.05).

**Figure 2:**
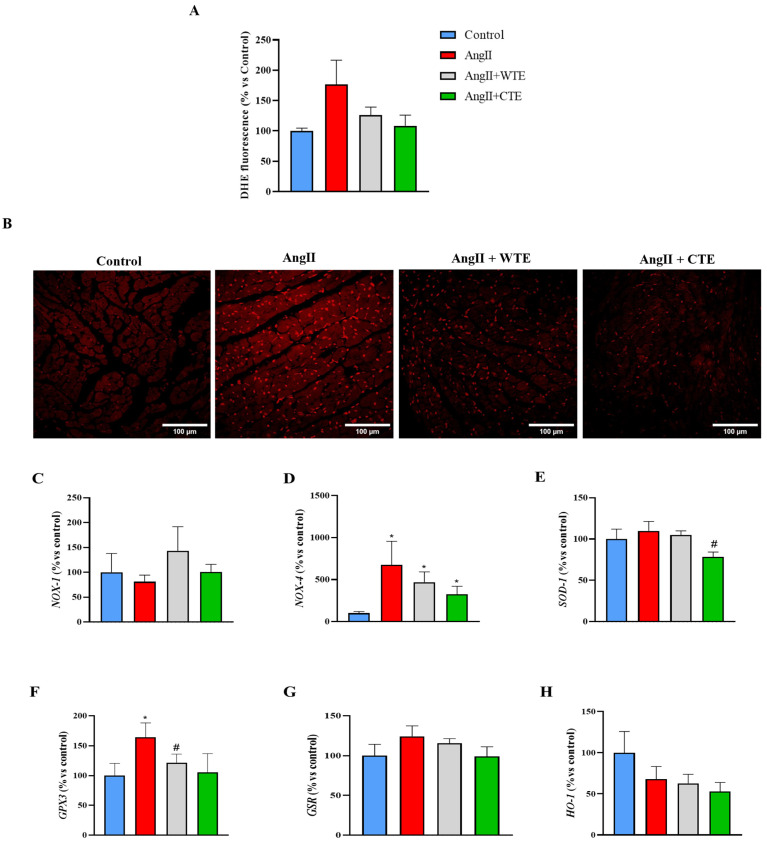
The quantification of reactive oxygen species (**A**) and representative images of heart sections stained with dihydroethidium (**B**). The scale bar is equivalent to 100 μm. The gene expression of NADPH oxidase 1 (**C**), NADPH oxidase 4 (**D**), superoxide dismutase 1 (**E**), glutathione peroxidase 3 (**F**), glutathione reductase (**G**), and Hemoxigenase 1 (**H**) in aorta of mice fed on a standard chow for 8 weeks and infused with saline for the last 4 weeks (control); mice fed on a standard chow for 8 weeks and infused with for the last 4 weeks (AngII); mice fed on a standard chow supplemented with White Tea Extract for 8 weeks and infused with AngII for the last 4 weeks (AngII + WTE); and mice fed on a standard chow supplemented with Complex Tea Extract for 8 weeks and infused with AngII for the last 4 weeks (AngII + CTE). Data are represented as mean value ± SEM, n = 8–10 mice/group, and expressed as a percentage vs. control; * *p* < 0.5 vs. control; # *p* < 0.05 vs. AngII.

The superoxide content and the mRNA levels of the main oxidative stress markers in myocardial tissue are shown in Figure 2. No differences were found in the content of superoxide anions between the heart sections of the animals of the different experimental groups (Figure 2A,B). Regarding the gene expression of oxidative stress markers, no significant differences were found between the different groups in the mRNA levels of *Nox-1*, *Gsr*, and *Ho-1* (Figure 2C, Figure 2G, and Figure 2H, respectively). Hypertensive mice showed an upregulation in the gene expression of *Nox-4* and *Gpx-3* compared to control mice (Figure 2D,F; *p* < 0.05 for both). Supplementation with the tea extracts did not prevent the AngII-induced overexpression of *Nox-4* in cardiac tissue. However, treatment with WTE significantly reduced the mRNA levels of *Gpx-3* and treatment with CTE downregulated the gene expression of *Sod-1* (Figure 2E; *p* < 0.05).

### 3.4. Effect of WTE and CTE Supplementation on Cardiac Apoptosis After IR

**Figure 3:**
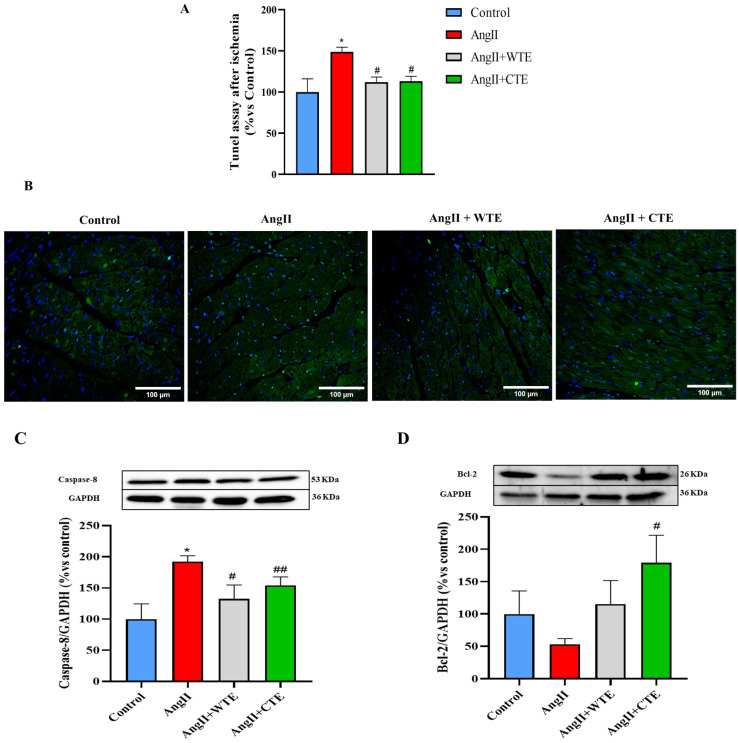
The quantification of cardiomyocyte apoptosis in ischemic hearts analyzed by TUNEL assay (**A**) and representative images of heart sections stained with TUNEL staining (**B**). The scale bar is equivalent to 100 μm. The protein content of Caspase 8 (**C**) and Bcl-2 (**D**) in ischemic hearts of mice fed on a standard chow for 8 weeks and infused with saline for the last 4 weeks (control); mice fed on a standard chow for 8 weeks and infused with AngII for the last 4 weeks (AngII); mice fed on a standard chow supplemented with White Tea Extract for 8 weeks and infused with AngII for the last 4 weeks (AngII + WTE); and mice fed on a standard chow supplemented with Complex Tea Extract for 8 weeks and infused with AngII for the last 4 weeks (AngII + CTE). Data are represented as mean value ± SEM, n = 8–10 mice/group, and expressed as a percentage vs. control; * *p* < 0.05 vs. control; # *p* < 0.05 vs. AngII; ## *p* < 0.01 vs. AngII.

The evaluation of cardiac apoptosis by TUNEL staining showed that supplementation with both WTE and CTE prevented the AngII-induced increase in DNA fragmentation in heart sections after IR (Figure 3A; *p* < 0.05 for WTE and *p* < 0.05 for CTE). In addition, AngII infusion increased the protein content of the pro-apoptotic protein caspase-8 (Figure 3C; *p* < 0.05 and Figure S1) and in decreased expression of the anti-apoptotic protein Bcl-2 (Figure 3D; *p* < 0.05 and Figure S2) in cardiac tissue. Supplementation with both tea extracts significantly reduced the AngII-induced overexpression of caspase 8 (*p* < 0.05 for WTE and *p* < 0.01 for CTE). However, only CTE treatment significantly increased Bcl2 protein expression in myocardial tissue (*p* < 0.05).

### 3.5. Vascular Function: Systolic Blood Pressure and Vascular Reactivity

**Figure 4:**
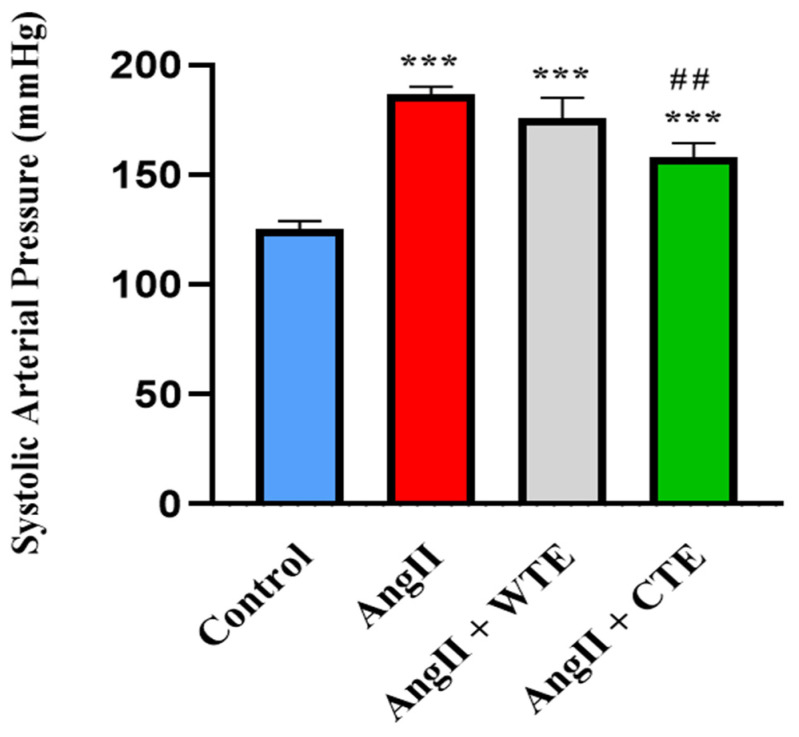
The systolic blood pressure of mice fed on a standard chow for 8 weeks and infused with saline for the last 4 weeks (control); mice fed on a standard chow for 8 weeks and infused with AngII for the last 4 weeks (AngII); mice fed on a standard chow supplemented with White Tea Extract for 8 weeks and infused with AngII for the last 4 weeks (AngII + WTE); and mice fed on a standard chow supplemented with Complex Tea Extract for 8 weeks and infused with AngII for the last 4 weeks (AngII + CTE). Data are represented as mean value ± SEM; n = 8–10 mice/group; *** *p* < 0.001 vs. control; ## *p* < 0.01 vs. AngII.

AngII infusion for 4 weeks produced a significant increase in systolic blood pressure (Figure 4 *p* < 0.001) that was attenuated only by supplementation with CTE (*p* < 0.01).

**Figure 5:**
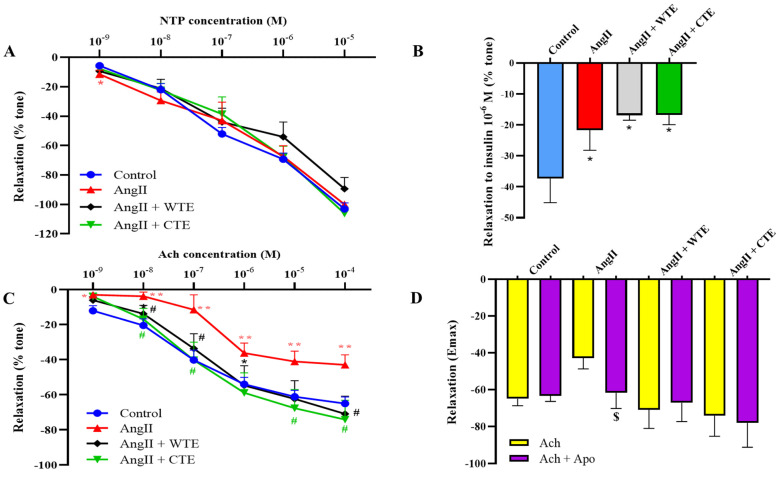
The relaxation of thoracic aortic segments to sodium nitroprusside (NTP) (10^−9^–10^−5^ M) (**A**), relaxation to insulin 10^−6^ M dose (**B**), relaxation to acetylcholine (ACh) (10^−9^–10^−4^ M) (**C**) and Emax relaxation to acetylcholine (10^−9^–10^−4^ M) in the presence/absence of apocynin (ACh/ACh + Apo) (10^−6^ M) (**D**) of mice fed on a standard chow for 8 weeks and infused with saline for the last 4 weeks (control); mice fed on a standard chow for 8 weeks and and infused with AngII for the last 4 weeks (AngII); mice fed on a standard chow supplemented with White Tea Extract for 8 weeks and infused with AngII for the last 4 weeks (AngII + WTE); and mice fed on a standard chow supplemented with Complex Tea Extract for 8 weeks and infused with AngII for the last 4 weeks (AngII + CTE). Data are represented as mean value ± SEM; n = 8–10 mice/group. * *p* < 0.05 vs. control; ** *p* < 0.01 vs. control; # *p* < 0.05 vs. AngII; $ *p* < 0.05 vs. Ach of its corresponding experimental group.

Endothelium-independent relaxation in response to sodium nitroprusside (NTP) was unchanged among experimental groups (Figure 5A), whereas the relaxation in response to a single dose of insulin was significantly lower in aorta segments from AngII-infused mice regardless of the supplementation with CTE or WTE (Figure 5B; *p* < 0.05 for all). However, the dose–response curve in response to acetylcholine showed a reduced relaxation in response to Ach in untreated hypertensive mice and therefore a lower endothelium-dependent relaxation at all doses studied compared to the control group (Figure 5C; *p* < 0.01). AngII-induced endothelial dysfunction was totally prevented by supplementation with both WTE and CTE (*p* < 0.05 for both). The reduced relaxation in response to Ach in untreated hypertensive mice was reversed by the incubation of aorta segments with the antioxidant apocynin. However, the presence of apocynin did not produce any changes in the relaxation to Ach in aorta segments compared to control or hypertensive mice supplemented with WTE or CTE (Figure 5D).

### 3.6. Effects of CTE and WTE on Arterial Wall Thickness

**Figure 6:**
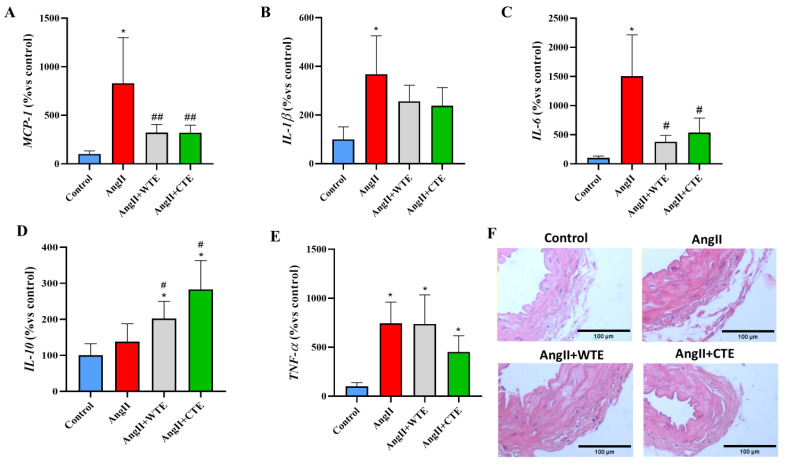
The arterial gene expression of Monocyte Chemotactic Protein-1 (**A**), interleukin-1β (**B**), interleukin-6 (**C**), interleukin-10 (**D**), and tumor necrosis factor α (**E**) and the H/E staining (**F**) of aorta rings of mice fed on a standard chow for 8 weeks and infused with saline for the last 4 weeks (control); mice fed on a standard chow for 8 weeks and infused with AngII for the last 4 weeks (AngII); mice fed on a standard chow supplemented with White Tea Extract for 8 weeks and infused with AngII for the last 4 weeks (AngII + WTE); and mice fed on a standard chow supplemented with Complex Tea Extract for 8 weeks and infused with AngII for the last 4 weeks (AngII + CTE). Data are represented as mean value ± SEM, n = 8–10 mice/group, and expressed as a percentage vs. control; * *p* < 0.05 vs. control; # *p* < 0.05 vs. AngII; ## *p* < 0.01 vs. AngII.

As shown in Figure 6F, AngII infusion induced a significant increase in the thickness of the tunica media that was prevented only by supplementation with CTE.

### 3.7. Effects of WTE and CTE on Inflammation and Oxidative Stress in Aortic Tissue

The gene expression of the main inflammatory markers in arterial tissue is shown in Figure 6. AngII infusion induced the overexpression of *Mcp-1*, *Il-1β*, *Il-6*, and *TNF-α* in aortic tissue (Figure 6A, Figure 6B, Figure 6C, and Figure 6E, respectively; *p* < 0.05 for all). Supplementation with both WTE and CTE prevented the AngII-induced upregulation of *Mcp-1* (*p* < 0.01) and Il-6 (*p* < 0.05) and significantly increased the mRNA levels of the anti-inflammatory cytokine *Il-10* (*p* < 0.01 for both).

**Figure 7:**
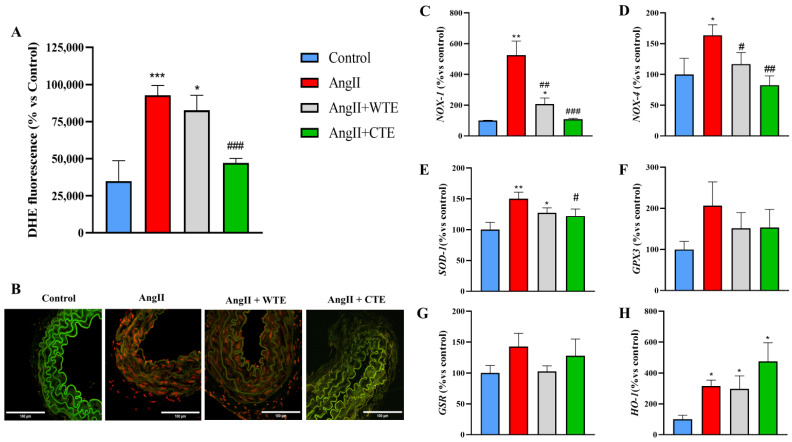
The quantification of reactive oxygen species (**A**) and representative images of aorta sections stained with dihydroethidium (**B**). The scale bar is equivalent to 100 μm. The gene expression of NADPH oxidase 1 (**C**), NADPH oxidase 4 (**D**), superoxide dismutase 1 (**E**), glutathione peroxidase 3 (**F**), glutathione reductase (**G**), and Hemoxigenase 1 (**H**) in aorta of mice fed on a standard chow for 8 weeks and infused with saline for the last 4 weeks (control); mice fed on a standard chow for 8 weeks and infused with AngII for the last 4 weeks (AngII); mice fed on a standard chow supplemented with White Tea Extract for 8 weeks and infused with AngII for the last 4 weeks (AngII + WTE); and mice fed on a standard chow supplemented with Complex Tea Extract for 8 weeks and infused with AngII for the last 4 weeks (AngII + CTE). Data are represented as mean value ± SEM, n = 8–10 mice/group, and expressed as a percentage vs. control; * *p* < 0.05 vs. control; ** *p* < 0.01 vs. control; *** *p* < 0.001 vs. control; # *p* < 0.05 vs. AngII; ## *p* < 0.01 vs. AngII; ### *p* < 0.001 vs. AngII.

The superoxide content and the mRNA levels of the main oxidative stress markers in arterial tissue are shown in Figure 7. Hypertension produced an increase in the content of superoxide anions in aortic tissue (Figure 7A,B; *p* < 0.001) along with increased mRNA levels of the prooxidant enzymes *Nox1* (Figure 7C; *p* < 0.01) and *Nox4* (Figure 7D; *p* < 0.05). Likewise, hypertension induced a significant increase in the mRNA levels of the antioxidant enzymes Sod1 (Figure 7E; *p* < 0.01) and *Ho-1* (Figure 7H; *p* < 0.001). Supplementation with both WTE and CTE significantly reduced the AngII-induced upregulation of Nox1 (*p* < 0. 01 for WTE and *p* < 0.001 for CTE) and *Nox4* (*p* < 0.05 for WTE and *p* < 0.01 for CTE). However, only supplementation with CTE prevented the increase in superoxide anion content in aortic sections (*p* < 0.001) and the AngII-induced overexpression of *Sod1* (*p* < 0.05).

### 3.8. Effects of CTE and WTE on the Components of the Renin–Angiotensin System

**Figure 8:**
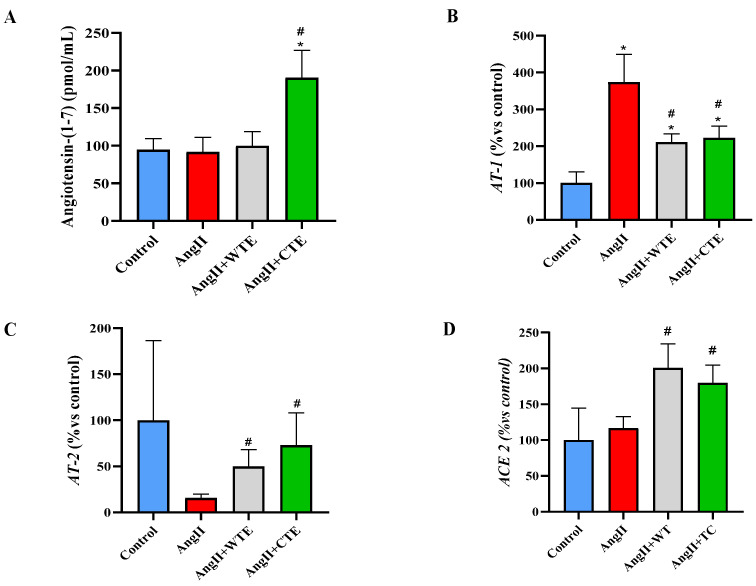
Plasma levels of angiotensin-(1-7) (**A**) and gene expression of angiotensin II receptor type 1 (**B**), angiotensin II receptor type 2 (**C**), and angiotensin I-converting enzyme 2 (**D**) in aortic tissue of mice fed on a standard chow for 8 weeks and infused with saline for the last 4 weeks (control); mice fed on a standard chow for 8 weeks and infused with AngII for the last 4 weeks (AngII); mice fed on a standard chow supplemented with White Tea Extract for 8 weeks and infused with AngII for the last 4 weeks (AngII + WTE); and mice fed on a standard chow supplemented with Complex Tea Extract for 8 weeks and infused with AngII for the last 4 weeks (AngII + CTE). Data are represented as mean value ± SEM, n = 8–10 mice/group, and expressed as a percentage vs. control; * *p* < 0.05 vs. control; # *p* < 0.05 vs. AngII.

To determine the effects of supplementation with tea extracts on the renin–angiotensin system, different parameters involved in this hormonal system were measured (Figure 8).

In the plasma, AngII-infused mice supplemented with CTE showed increased circulating levels of the peptide angiotensin 1-7 compared to control and untreated hypertensive mice (*p* < 0.05 for both). Moreover, hypertensive mice showed an increase in the gene expression *At1r* (*p* < 0.05) in aortic tissue (*p* < 0.05). Supplementation with both tea extracts reduced the AngII-induced increase in *At1r* (*p* < 0.05 for both). Moreover, treatment with both CTE and WTE significantly increased the mRNA levels of *At2r* and *Ace2* in arterial tissue (*p* < 0.05).

### 3.9. Antioxidant Capacity of CTE and WTE and Plasma Levels of Polar Metabolites and MDA

The analyses of polar metabolites by HPLC, together with an assessment of the malondialdehyde (MDA) content and antioxidant capacity (DPPH assay) in plasma samples from the different groups, are depicted in Table 3. Plasma samples from control mice showed higher concentrations in polar metabolites compared to mice infused with AngII (*p* < 0.001). However, mice treated with the tea extracts showed a significant increase in the polar metabolites content in plasma (about two folds for AngII + CTE and over three folds for the AngII + WTE group) compared to the AngII group. A similar trend was observed for the antioxidant capacity which was significantly decreased in the plasma of AngII-infused mice compared to controls and increased in AngII-infused mice supplemented with both CTE and WTE, although no significant differences were observed among groups. Regarding MDA content in plasma, no significant differences were observed among experimental groups (Table 3).

## 4. Discussion

In this study, we report, for the first time, the positive effects of two standardized tea extracts, one of white tea (WTE), and another one of green and black tea (CTE), on the cardiovascular alterations induced by AngII in mice. The beneficial effects on cardiovascular function are also accompanied by modifications in body composition. In particular, supplementation with both WTE and CTE induced a significant decrease in body weight and in the amount of visceral and subcutaneous adipose tissue. These results agree with a previous study reported by our group in which the supplementation of mice with CTE with metabolic syndrome significantly reduces body weight gain and adiposity due to a direct antiadipogenic effect [31].

In the heart, AngII infusion induced a significant increase in coronary pressure before IR. This increase in coronary pressure was expected since AngII exerts a potent vasoconstrictor effect through its binding to the AT1R [38]. The AngII-induced vasoconstrictor effect was not prevented by CTE supplementation. However, supplementation with WTE significantly reduced the AngII-induced increase in coronary pressure, pointing to a vasodilator effect of WTE on coronary circulation. These results agree with a very recent study in which Anji white tea exerted a direct vasodilator effect in several vascular beds, including the coronary one. However, as in our study, this effect is not produced by (-)-epigallocatechin gallate (EGCG), the main bioactive component present in green tea [39].

In addition to the effects on coronary pressure, one of the most relevant results of this study is that supplementation with both tea extracts, WTE and CTE, increased heart contractility after IR, with these effects being associated with decreased apoptosis of cardiomyocytes. This is consistent with previous studies in which the administration of a green tea extract significantly improved myocardial contractility in a context of ischemia, both in vitro [40] and in vivo [41]. The cardioprotective effects of green tea in the context of ischemia have already been reported and seem to be mediated, at least in part, by the presence of ECGC [42]. Indeed, the direct administration of ECGC before ischemia and during reperfusion results in decreased myocardial damage [43]. Likewise, oral supplementation with ECGC improved the recovery of cardiac function after IR in rats due to a decrease in oxidative stress and apoptosis [44]. Moreover, ECGC prevented isoproterenol-induced myocardial apoptosis through the inhibition of the intrinsic apoptotic pathway [45]. As previously described [46], the antiapoptotic effects of CTE found in this study seem to be related to an inhibition of the intrinsic apoptotic pathway since CTE supplementation significantly increased the content of the antiapoptotic protein Blc-2 in myocardial tissue [47]. However, supplementation with both tea extracts also decreased the AngII-induced overexpression of caspase-8, also demonstrating an inhibitory effect on the apoptotic extrinsic pathway.

To further investigate the mechanisms by which supplementation with CTE or WTE improve cardiac function after IR and taking into account the antioxidant [23] and anti-inflammatory effects of tea [48], we analyzed the gene expression of the main markers of oxidative stress and inflammation in myocardial tissue. We did not find significant changes, either in the content of superoxide anion or in the cardiac mRNA levels of the main inflammatory and oxidative stress markers, except for a significant upregulation of *Nox4* and *Gpx3* in response to AngII infusion that was attenuated, in the case of *Gpx3*, by WTE supplementation. Moreover, supplementation with CTE decreased the cardiac mRNA levels of *Sod1*. These results disagree with other studies in which supplementation with white or green tea exerts antioxidant effects in myocardial tissue, both in physiological conditions [49] and in different pathological situations such as oxidative injury induced by adriamycin [50], diabetes [51], and aging [52]. Likewise, the oral administration of ECGC for two weeks exerted cardioprotective effects in rat hearts subjected to IR by reducing oxidative stress [44]. However, in a previous study from our group, we did not find significant changes in the gene expression of oxidative stress markers in ischemic hearts from mice with metabolic syndrome [30]. The discrepancies among the different studies may be due to differences in experimental models, species, tea composition, ways of administration, and treatment duration.

As with the oxidative stress markers, no differences were found among experimental groups in the cardiac mRNA levels of proinflammatory cytokines, except for a downregulation of *Il-6* in response to WTE supplementation. This result agrees with a previous study in which white tea is reported to exert a potent anti-inflammatory effect in acute and chronic experimental models of inflammation [48].

At the vascular level, the results of the in vivo study showed that AngII infusion resulted in a significant increase in systolic blood pressure (SBP), as has been widely described in both experimental animals [53] and in humans [54]. Supplementation with WTE for 8 weeks did not reduce AngII-induced hypertension. This result disagrees with our previous study, in which supplementation with the same white tea extract significantly decreased SBP in mice with metabolic syndrome [30]. The discrepancy between both studies is most likely due to differences in the experimental models (AngII infusion vs. metabolic syndrome), since AngII infusion produces more severe hypertension than metabolic syndrome. Furthermore, the lower duration of treatment (8 weeks vs. 20 weeks) may also have an impact on the absence of antihypertensive effects in the AngII infusion model.

Opposite to WTE, our results also show that CTE supplementation significantly reduced SBP in mice with angiotensin II-induced hypertension. The positive effects of CTE and the lack of effects of WTE on AngII-induced hypertension are most likely due to the different composition of both extracts, and particularly to differences in the polyphenol content. Indeed, we have previously reported that CTE contains a higher amount of bioactive compounds, such as gallic acid, xanthines and flavan-3-ols, both monomeric and oligomeric, than WTE extract [30].

The positive antihypertensive effects of CTE are not only in agreement with those found in our previous study [30], but also with those reported by other authors. Particularly, green and black tea consumption are reported to decrease systolic blood pressure in different models of hypertension such as angiotensin II-induced hypertension [55], stroke-prone spontaneously hypertensive rats [25], and in a rat model of salt-induced hypertension [56]. The antihypertensive effects of CTE are most likely due to the presence of catechins, since the direct administration of epigallocatechin gallate is reported to decrease blood pressure in spontaneously hypertensive rats [57]. Likewise, in humans, several population-based studies and meta-analyses of randomized controlled trials have associated the higher tea intake with lower blood pressure in both normotensive and hypertensive subjects [58,59,60].

To further investigate the effects of both CTE and WTE on vascular function, we analyzed the thickness of the aorta tunica media and performed experiments of vascular reactivity in aorta segments of mice from the different experimental groups. Only supplementation with CTE attenuated the Ang-II increase in arterial wall thickness, an effect that may be the result of reduced vascular smooth muscle cell (VSMC) migration, as has been previously reported [61].

CTE or WTE did not induce significant differences either in the endothelium-independent relaxation nor in the vascular response to insulin. However, supplementation with both tea extracts improved endothelium-dependent relaxation, thus attenuating AngII-induced endothelial dysfunction. Moreover, the incubation of aorta segments with the NADPH inhibitor apocynin prevented the reduced endothelium-dependent relaxation in aorta segments from untreated AngII-infused mice, demonstrating that AngII-induced endothelial dysfunction was the result of decreased NO due to increased oxidative stress, as previously described [62]. On the contrary, preincubation with the apocynin did not modify the endothelium-dependent relaxation, either in aorta segments from control mice or in aorta segments from hypertensive mice supplemented with WTE or CTE, which demonstrates that the positive effects of both tea extracts on endothelial dysfunction are due, at least in part, to their antioxidant effects. This was confirmed by the assessment of the superoxide content and the gene expression of the main oxidative markers in arterial tissue. Both CTE and WTE supplementation prevented the AngII-induced overexpression of the prooxidant enzymes Nox1 and Nox4, demonstrating their antioxidant effects. However, only supplementation with CTE significantly reduced the content of superoxide anion and the mRNA levels of the superoxide neutralizing enzyme Sod1 in arterial tissue, demonstrating the higher antioxidant activity of CTE compared to WTE. Nevertheless, both extracts seem to exert similar effects on inflammation since both treatments significantly reduce the arterial gene expression of Mcp-1 and Il-6. Thus, the effects of CTE treatment in reducing AngII-induced hypertension seem to be more related to its antioxidant effect rather than its anti-inflammatory effect. These results are consistent with our previous study in which both tea extracts reduced endothelial dysfunction and the mRNA levels of inflammatory and oxidative stress markers in arterial tissue in mice with metabolic syndrome [30]. Likewise, the administration of a green tea extract to rats with AngII-induced hypertension improved endothelial dysfunction by decreasing the production of ROS [63]. Furthermore, treatment with phenolic compounds produced similar results, preventing both endothelial dysfunction and hypertension [64].

In addition to the antioxidant and anti-inflammatory effects, the beneficial effects of both tea extracts on vascular function, and particularly on endothelial function, may also be related to their effects in terms of modulating the renin–angiotensin system. Indeed, our results show that supplementation with both CTE and WTE prevents the AngII-induced upregulation of *At1r* and the AngII-induced downregulation of *At2r* in arterial tissue. These effects may have a positive impact on the improvement of endothelial function since AngII binding to AT1R promotes vasoconstriction, inflammation, and oxidative stress, whereas the activation of the AT2R receptors exerts opposite effects [65]. These results are consistent with previous in vivo studies in which supplementation with a green tea extract significantly inhibits the activation of RAAS in an experimental model of nephrotoxicity [66]. Moreover, supplementation with EGCG attenuates the development of hypertension in spontaneously hypertensive rats by modulating RAS activation [67]. These results are more likely associated with the ability of tea to inhibit the ACE activity both in vitro [68,69] and in vivo [70]. However, treatment with wine polyphenols prevented the development of hypertension in spontaneously hypertensive rats without affecting the expression of *At1r*. The discrepancies between these studies are most likely due to differences in the extract compositions and/or in the duration of treatments.

In addition to *At1r* and *At2r*, we also analyzed the mRNA levels of *Ace2* in arterial tissue as well as the circulating concentrations of the vasodilator peptide angiotensin 1-7 (Ang 1-7) as the binding of AngII to AT1R is reported to reduce *Ace2* expression/activation in the context of hypertension [71]. Opposite to the results reported by Igase et. al, we did not find significant changes in the mRNA levels of *Ace2* between control and AngII-infused mice in arterial tissue, which may be explained by differences in species (mice vs. rats) and/or experimental models (SHR vs. AngII infusion). However, we did find an overexpression of *Ace2* in arterial tissue from hypertensive mice supplemented with both CTE and WTE. However, only CTE supplemented mice showed a significant increase in the plasma levels of Ang 1-7. These results, together with the decreased levels of superoxide anion in arterial tissue and the attenuation of AngII-induced arterial remodeling may explain the significant reduction in SBP after treatment with CTE but not with WTE. The stronger antihypertensive effect of CTE compared to WTE is most likely related to differences in the concentration of bioactive compounds between both tea extracts since, as previously described, CTE is more concentrated on gallic acid, xanthines, and flavan-3-ols than WTE. Moreover, CTE contains both monomeric and oligomeric flavan-3-ols (theaflavins), whereas WTE is just composed of monomeric flavan-3-ols [30].

## 5. Conclusions

In conclusion, the results of this study indicate that supplementation with both CTE and WTE prevents some of the cardiovascular alterations associated with AngII infusion in mice due to their anti-inflammatory and antioxidant effects. However, only CTE supplementation significantly reduces SBP, with this effect most likely being related to its ability to reduce arterial remodeling and the amount of superoxide anions in arterial tissue and to increase the circulating levels of the vasodilator peptide Ang 1-7. In general, these results demonstrate the potential of supplementation with these tea extracts to support cardiovascular health.

The main limitations of the study are the following: First, the study is based on an experimental model of angiotensin II-induced hypertension in mice, which may not fully replicate the complexities of cardiovascular alterations associated with human hypertension. Second, the duration of supplementation with tea extracts was limited to eight weeks, which potentially failure to capture long-term effects. Finally, as we used an inbred strain of mice, the potential influence of genetic variability in response to supplementation with this specific tea extract was not considered. Thus, future research studies should explore the long-term effects of tea extract supplementation in diverse hypertension models and human clinical trials. Additionally, assessing longer durations and the role of genetic variability in response to tea extracts may help to enhance personalized therapeutic applications.

## Figures and Tables

**Table 1:** Body weight, total food intake, and organ weights of mice fed on a standard chow for 8 weeks and infused with saline for the last 4 weeks (control), mice fed on a standard chow for 8 weeks and infused with AngII with for the last 4 weeks (AngII), mice fed on a standard chow supplemented with White Tea Extract for 8 weeks and infused with AngII for the last 4 weeks (AngII + WTE), and mice fed on a standard chow supplemented with Complex Tea Extract for 8 weeks and infused with AngII for the last 4 weeks (AngII + CTE).

	Control	AngII	AngII + WTE	AngII + CTE
Body weight (g)	28.9 ± 0.6	28.5 ± 0.6	24.8 ± 0.7 ***###	26.1 ± 0.6 **##
Total food intake (g)	153.9 ± 6.6	164.2 ± 6.5	123.2 ± 7.8 *##	123.2 ± 6.5 ##
Heart (mg/cm)	104.7 ± 5.0	129.4 ± 5.4 **	112.9 ± 3.4 #	118.1 ± 6.7 *#
Spleen (mg/cm)	40.1 ± 2.1	41.8 ± 3.0	29.8 ± 1.8 **##	31.9 ± 1.1 **##
Liver (mg/cm)	641.6 ± 33.2	598.9 ± 16.1 *	483.3 ± 49.3 *#	559.3 ± 48.7
Epidydimal visceral adipose tissue (mg/cm)	222.7 ± 17.1	186.5 ± 17.5	120.6 ± 15.7 ***##	132.0 ± 15.2 ***#
Retroperitoneal visceral adipose tissue (mg/cm)	52.9 ± 6.9	48.1 ± 5.2	28.0 ± 3.4 **##	28.8 ± 3.4 **##
Lumbar subcutaneous adipose tissue (mg/cm)	99.5 ± 12.1	85.6 ± 10.6	73.4 ± 11.7	62.2 ± 5.5 *#
Interscapular brown adipose tissue (mg/cm)	55.3 ± 6.1	49.5 ± 5.5	49.8 ± 4.7	52.4 ± 4.2
Kidneys (mg/cm)	150.9 ± 4.0	141.8 ± 6.4	123.5 ± 3.3 ***#	124.2 ± 4.1 ***#
Adrenal glands (mg/cm)	1.4 ± 0.1	1.7 ± 0.1 *	1.7 ± 0.1 *	1.7 ± 0.1 *
Brain (mg/cm)	247.0 ± 2.2	241.9 ± 2.0	237.5 ± 2.4 *	237.8 ± 3.7 *
Gastrocnemius (mg/cm)	77.1 ± 3.3	72.3 ± 3.4	69.0 ± 2.3 *	70.8 ± 3.5

Data are represented as mean ± SEM; n = 8–10 mice/group. * *p* < 0.05 vs. control; ** *p* < 0.01 vs. control; *** *p* < 0.001 vs. control; # *p* < 0.05 vs. AngII; ## *p* < 0.01 vs. AngII; ### *p* < 0.001 vs. AngII.

**Table 2:** Coronary pressure and heart contractility (dp/dt) in hearts subjected to ischemia-reperfusion (IR) of mice fed on a standard chow for 8 weeks and infused with saline for the last 4 weeks (control), mice fed on standard chow for 8 weeks and infused with AngII with for the last 4 weeks (AngII), mice fed on a standard chow supplemented with White Tea Extract for 8 weeks and infused with AngII for the last 4 weeks (AngII + WTE), and mice fed on a standard chow supplemented with Complex Tea Extract for 8 weeks and infused with AngII for the last 4 weeks (AngII + CTE).

	**Coronary Pressure (mmHg)**	
	**Before IR**	**After IR**
Control	67.8 ± 3.2	75.8 ± 2.3
AngII	88.4 ± 1.5 *	80.4 ± 0.8
AngII + WTE	76.7 ± 1.1 #	86.4 ± 1.9
AngII + CTE	82.1 ± 1.8	81.4 ± 0.9
	**Heart Contractility (*dp/dt*)**	
	**Before IR**	**After IR**
Control	2137.6 ± 117.3	1095.5 ± 133.5
AngII	1812.7 ± 106.6	1591.1 ± 85.6
AngII + WTE	2123.6 ± 88.6	2319.1 ± 135.6 *#
AngII + CTE	2417.9 ± 80.5	2183.4 ± 81.1 **#

Data are represented as mean ± SEM; n = 8–10 mice/group. * *p* < 0.05 vs. control; ** *p* < 0.01 vs. control; # *p* < 0.05 vs. AngII.

**Table 3:** Polar metabolites, malondialdehyde (MDA) content, and antioxidant capacity (DPPH assay) analyses in plasma of mice fed on a standard chow for 8 weeks and infused with saline for the last 4 weeks (control); mice fed on standard chow for 8 weeks and infused with AngII for the last 4 weeks (AngII); mice fed on a standard chow supplemented with White Tea Extract for 8 weeks and infused with AngII for the last 4 weeks (AngII + WTE); and mice fed on a standard chow supplemented with Complex Tea Extract for 8 weeks and infused with AngII for the last 4 weeks (AngII + CTE).

	Control	AngII	AngII + WTE	AngII + CTE
Polar metabolites (HPLC, area units/10 µL plasma)	712.6 ± 10.3	140.1 ± 7.6 ***	311.3 ± 4.1 ***###	466.9 ± 7.5 ***###$$
MDA (nmol/mL plasma)	0.7 ± 0.2	1.2 ± 0.3	0.7 ± 0.2	0.6 ± 0.4
DPPH (µM/µL plasma)	2.07 ± 0.75	0.85 ± 0.35 *	0.64 ± 0.29 **	1.39 ± 0.44

Data are represented as mean value ± SEM, n = 8–10 mice/group, and expressed as a percentage vs. control; * *p* < 0.05 vs. control; ** *p* < 0.01 vs. control; *** *p* < 0.001 vs. control; ### *p* < 0.001 vs. AngII; $$ *p* < 0.001 vs. AngII + WTE.

## Data Availability

Data are contained within the article.

## Supplementary Materials

The following supporting information can be downloaded at: https://www.mdpi.com/article/10.3390/antiox14010047/s1, Figure S1: Original Western blots of caspase-8 and GAPDH in ischemic hearts from mice fed on a standard diet for 8 weeks and infused with saline for the last 4 weeks (control); mice fed on a standard diet for 8 weeks and infused with AngII for the last 4 weeks (AngII); mice fed on a standard diet supplemented with white tea extract for 8 weeks and infused with AngII for the last 4 weeks (AngII + WTE); and mice fed on a standard diet supplemented with complex tea extract for 8 weeks and infused with AngII for the last 4 weeks (AngII + CTE). Molecular weight marker was obtained from colorimetric imaging and original blots obtained from chemiluminescent imaging. Dotted and dashed lines correspond to the same gel developed first with Caspase 8 and subsequently with GAPDH. The dashed lines indicate the image shown in Figure 3C; Figure S2: Original Western blots of Bcl2 and GAPDH in ischemic hearts from mice fed on a standard diet for 8 weeks and infused with saline for the last 4 weeks (control); mice fed on a standard diet for 8 weeks and infused with AngII for the last 4 weeks (AngII); mice fed on a standard diet supplemented with white tea extract for 8 weeks and infused with AngII for the last 4 weeks (AngII + WTE); and mice fed on a standard diet supplemented with complex tea extract for 8 weeks and infused with AngII for the last 4 weeks (AngII + CTE). Molecular weight marker was obtained from colorimetric imaging and original blots obtained from chemiluminescent imaging. Dotted and dashed lines correspond to the same gel developed first with Caspase 8 and subsequently with GAPDH. The dashed lines indicate the image shown in Figure 3D.
