# Supplementary figures and images for: Supplementation with Standardized Green/Black or White Tea Extracts Attenuates Hypertension and Ischemia-Reperfusion-Induced Myocardial Damage in Mice Infused with Angiotensin II
Source: Antioxidants (Basel). 2025 Jan 3;14(1):47. doi: 10.3390/antiox14010047 (PMC11762166; doi:10.3390/antiox14010047)

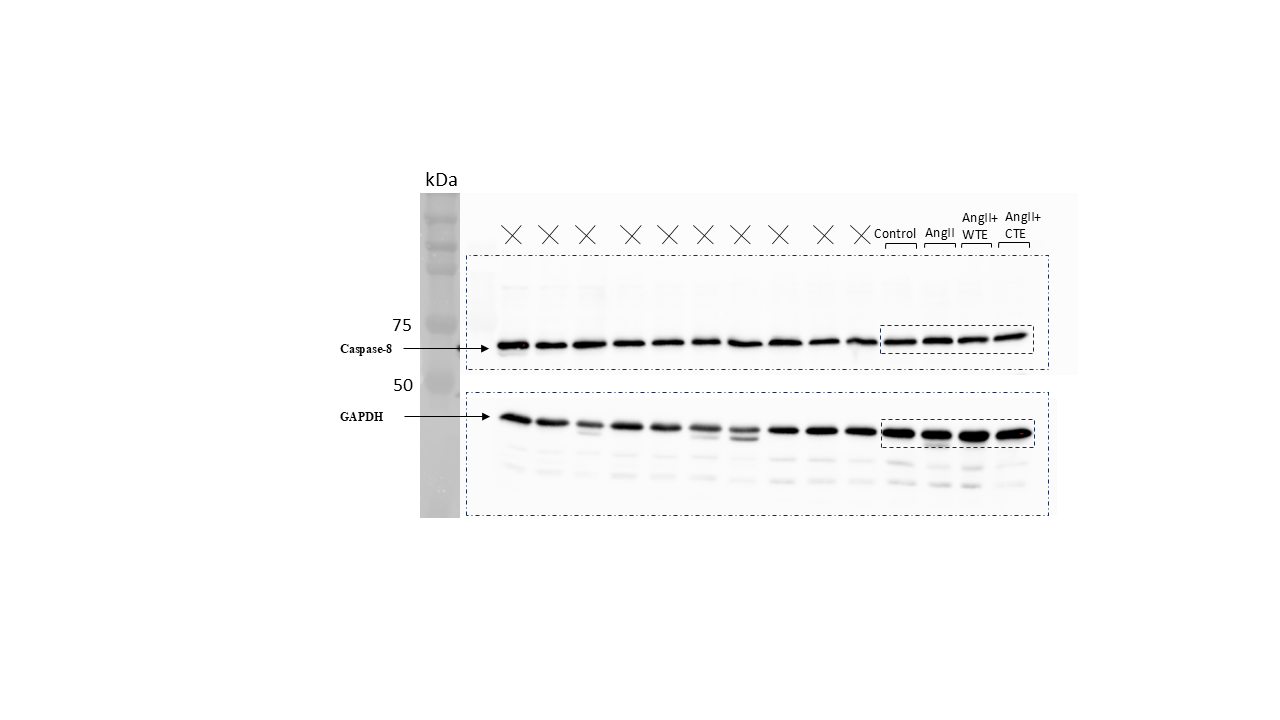

Supplement: Supplementary file 1 [file antioxidants-14-00047-s001.zip › Figure S1.tif]

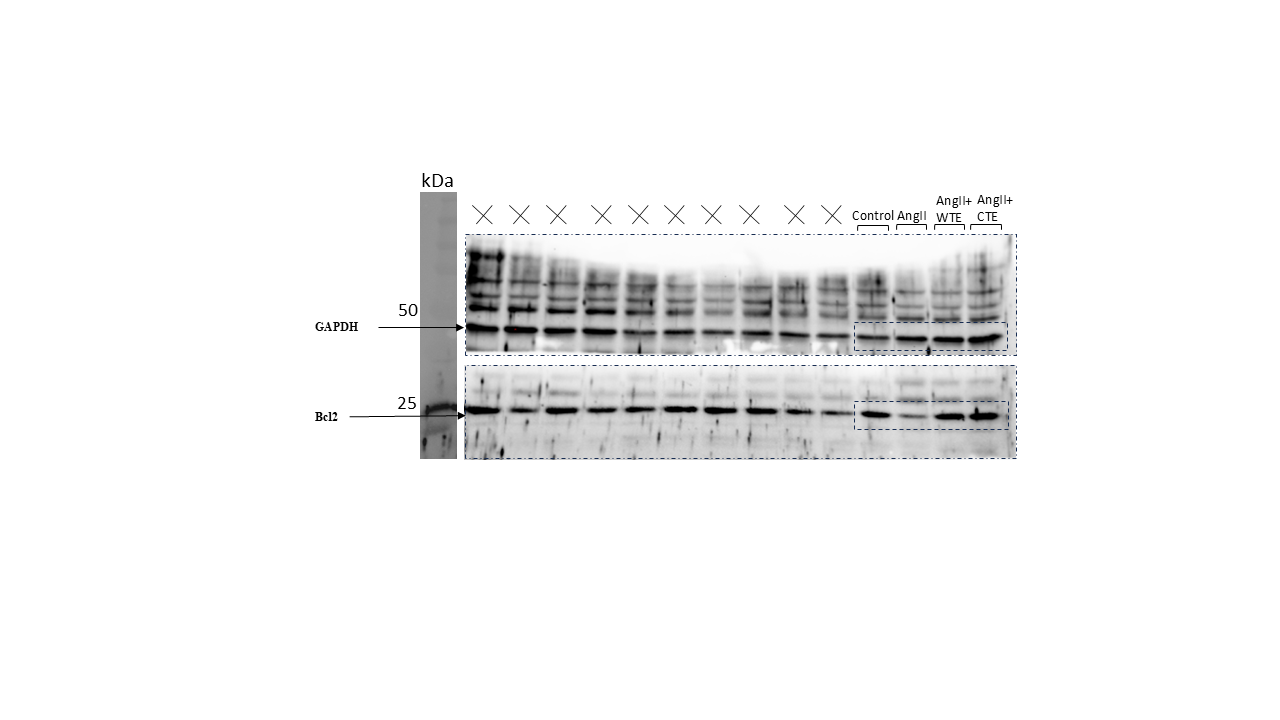

Supplement: Supplementary file 1 [file antioxidants-14-00047-s001.zip › Figure S2.tif]
